# Reporter pathway analysis from transcriptome data: Metabolite-centric versus Reaction-centric approach

**DOI:** 10.1038/srep14563

**Published:** 2015-09-28

**Authors:** Tunahan Çakır

**Affiliations:** 1 Gebze Technical University, Department of Bioengineering, 41400, Gebze, Kocaeli, Turkey

## Abstract

A systems-based investigation of the effect of perturbations on metabolic machinery is crucial to elucidate the mechanism behind perturbations. One way to investigate the perturbation-induced changes within the cell metabolism is to focus on pathway-level effects. In this study, three different perturbation types (genetic, environmental and disease-based) are analyzed to compute a list of reporter pathways, metabolic pathways which are significantly affected from a perturbation. The most common omics data type, transcriptome, is used as an input to the bioinformatic analysis. The pathways are scored by two alternative approaches: by averaging the changes in the expression levels of the genes controlling the associated reactions (reaction-centric), and by averaging the changes in the associated metabolites which were scored based on the associated genes (metabolite-centric). The analysis reveals the superiority of the novel metabolite-centric approach over the commonly used reaction-centric approach since it is based on metabolites which better represent the cross-talk among different pathways, enabling a more global and realistic cataloguing of network-wide perturbation effects.

Pathways are functional units which perform certain cellular tasks. A practical way to grasp the effect of a perturbation on molecular mechanisms, whether it is environmental, genetic or disease-based, is to check how it affects hundreds of cellular pathways. The availability of omics data facilitates the documentation of the perturbation-induced changes in the cell via the pathways constituting its cellular networks. Metabolic pathways are one of the most appealing pathway types in this sense since metabolism is the engine that runs the cellular factory. Several computational methods emerged, for example, to integrate transcriptome data and metabolic networks to catalogue how the pathways respond to a perturbation^1^,^2^,^3^,^4^.

The most common pathway-oriented approach to bridge mRNA-level changes and observed phenotypes is pathway enrichment, which statistically analyze the enrichment of associated pathways for a set of genes found to be significantly affected from the perturbation, without considering the magnitudes of individual changes observed for the gene transcripts^5^. However, it is also crucial to consider the information on the connectivity and the magnitude of the significance of change for such differentially expressed genes. More importantly, genes not differentially expressed should also be accounted since they can exhibit significant coordinated changes when considered together^6^. Therefore, metabolic networks can be used as a scaffold to map the differential expression information of all genes such that pathway-level perturbation effects are presented by utilizing network connectivity^7^,^8^. Organism-specific metabolic network information for such an analysis can be obtained from popular pathway databases such as KEGG^9^, BioCyc^10^ and Reactome^11^, or it is available via curated genome-scale metabolic models^12^.

Here I present a new approach to catalogue metabolic pathway-level perturbation effects based on transcriptome data. Approaches in the literature for the determination of perturbed metabolic pathways are reaction-centric, i.e. pathways are scored based on the expression levels of the genes controlling the associated reactions. A metabolite-centric approach which takes into account all the reactions which consume or produce the metabolites of the pathway is needed to better represent the effects of a perturbation on network-level. A novel metabolite-centric scoring algorithm is employed to integrate organism-specific metabolic networks with the statistical changes on the genes which control the reactions functioning in the network, and it is compared to the more straightforward reaction-centric algorithm. The algorithm builds on the reporter metabolite approach commonly used in the analysis of transcriptome-associated regulations in metabolic networks^7^. The metabolite-centric reporter pathway analysis (RPA^m^) is shown to outperform the reaction-centric scoring of pathways (RPA^r^) in terms of reporting perturbation-associated underlying mechanisms.

## Results and Discussion

### Reporter pathways for a Genetic Perturbation

GDH1 is known to encode NADPH-dependent conversion of 2-ketoglutarate to glutamate in the yeast *S. cerevisiae*. A deletion was introduced to this gene to perturb the cofactor balance within the yeast cells^13^. The resulting strain had defects in ammonia assimilation, and had decreased glycerol production and increased ethanol production. The corresponding transcriptome data was later analyzed based on differential statistical analysis, however the analysis of a small subset of data that cover only genes controlling cofactor-associated enzymes, not the genome-wide analysis, revealed results which can be biologically linked to the source of perturbation^14^. Later, reporter metabolite analysis of the same data enabled a noticeably better deduction of the mechanism of the genetic perturbation^7^. They have identified few metabolites involved in the pentose phosphate pathway as reporters, which is reasonable since this pathway is a source of NADPH. This showed the power of the approaches which take into account network connectivity and do not make a priori selection on the genes based on their significance.

Here, I analyze the same dataset by the reporter pathway approach. Reporter pathways were calculated based on the two alternative approaches, RPA^r^ and RPA^m^, and also by PADOG. In addition to the NADPH imbalance, the deletion also results in an NADH imbalance in the cell since the deleted reaction is replaced by an NADH-dependent route by the cell. Therefore, pathways linked to NADPH and NADH are expected to be perturbed. The RPA^m^ results revealed 35 significant pathways affected from the perturbation (Table 1). Among those are mainly pentose phosphate pathway, pathways of branched-chain amino acids (leucine, isoleucine and valine), glycolysis/gluconeogenesis, TCA cycle and NADPH/NADH interconversion pathways, all directly related to the source of perturbation. The deletion of GDH1 gene forces the NADH-dependent route to be active, leading to the changes in all these NADH or NADPH dependent pathways. A study on the metabolome analysis of a similar strain with GDH1 deletion and GDH2 overexpression reports statistically significant changes on the branched chain amino acids valine, leucine and isoleucine, as well as on lysine^15^. Several related pathways were predicted to be significantly dysregulated in the RPA^m^ analysis. RPA^m^ could identify a significant change in the direct source of perturbation, 2-ketoglutarate dehydrogenase complex, successfully. RPA^r^, on the other hand, could not capture the significant change in this pathway. Moreover, glycolytic pathway, lysine biosynthesis and other pathways which are linked to the perturbation were not identified in the RPA^r^ analysis. In a study reporting a mutant *S. cerevisiae* strain with enhanced NADPH demand, the change in glycerol production was explained by a change in folate cycle^16^. RPA^m^ reports significant changes in folate cycle related pathways in parallel to this hypothesis, which could not be attained by RPA^r^. Pentose phosphate pathway, for example, was ranked as top fourth pathway in RPA^m^ analysis whereas it was the 13^th^ most significant pathway in RPA^r^ analysis. Besides, non-oxidative pentose phosphate pathway was only identified by RPA^m^.

PADOG results were fairly stringent, leading to only few significant pathways: TCA cycle and leucine biosynthesis. The pathways pentose phosphate pathway and 2-ketoglutarate dehydrogenase complex were identified among the top five affected pathways, albeit with a p-value of 0.04. As mentioned in the methods section, for all calculations, the most significant gene was first mapped to the corresponding reaction if the reaction is associated with multiple genes. PADOG calculations were also repeated by using all genes associated with a pathway without making this initial mapping. Interestingly, assigned significance levels for TCA cycle and pentose phosphate pathways were 0.11 and 0.32 this time, respectively.

The results show the power of metabolite-centered reporter pathway analysis since the approach led to the identification of a number of significantly affected pathways all relevant to the genetic perturbation, which was hardly identified in the initial analysis of the dataset^14^, and revealed to some extent in the original paper introducing the reporter metabolite analysis^7^. Reporter metabolite analysis identifies metabolites around which most significant transcriptional changes occur. RPA^m^, on the other hand, relying upon the strength of reporter metabolite analysis, takes it into a new level since it is easier to interpret the scores of pathway activity information than metabolite scores.

### Reporter Pathways for an Environmental Perturbation

A commonly studied environmental perturbation in yeast *S. cerevisiae* is based on the availability of oxygen. Aerobic and anaerobic cultures have been analyzed in transcriptome level to enlighten underlying mechanisms^17^. RPA^m^ and RPA^r^ analyses both led to the identification of aerobic respiration (electron transport chain), one of the major related pathways directly related to the source of perturbation (Table 2). A major distinctive characteristics of this environmental perturbation is the activation of electron transport chain in aerobic conditions. Another commonly identified pathway, glycolysis, is in line with the findings that glycolytic fluxes were several folds higher^18^ and the concentrations of major glycolytic metabolites changed in anaerobic condition^19^. There is also an increase reported in the mRNA levels of gluconeogenesis specific genes, FBP1 and PCK1 in aerobiosis^19^, in accordance with the detection of this pathway as reporter by both RPA^m^ and RPA^r^.

The increase in glycolytic flux necessitates a decrease in the pentose phosphate pathway flux since they are competing branches in the metabolic network. This was reported at flux level^20^, at proteome level^21^, and also at mRNA level^19^,^22^. The metabolites of the pathway also exhibited changes between aerobic and anaerobic conditions^19^. As obvious, the activity of TCA cycle is minimal in anaerobic chemostat cultures since the flux is directed towards mostly ethanol whereas a fully active TCA cycle is observed in aerobic chemostat cultures since there is no byproduct formation. The pathway also showed alterations in the metabolome level^19^. The dysregulation of TCA cycle and pentose phosphate pathway in response to oxygen availability was only captured by RPA^m^ (Table 2).

Several fatty-acid degradation pathways appear in Table 2, including heptadecenoyl-CoA and octadecadienoyl-CoA degradation and oleate oxidation. Fatty acid oxidation is known to be downregulated at anaerobiosis^22^. On the other hand, in anaerobic conditions, the yeast needs to be supplemented by oleate and sterols since the synthesis of these biomolecules are oxygen-dependent^23^,^24^. Therefore an alteration is expected in the related pathways from aerobiosis to anaerobiosis, explaining the identification of oleate oxidation and zymmosterol biosynthesis as reporter pathways (Table 2). In addition to sterol and fatty acid biosynthesis, heme biosynthesis pathway is also oxygen dependent^25^,^26^. The presence of heme activates the transcription factor Hap1p (heme activated protein) which is responsible for the expression of a set of genes involved in respiration^25^,^27^. This mechanism was pointed out by both RPA^m^ and RPA^r^ since heme biosynthesis was in the list. Another identified pathway, known also as kynurenine pathway, is the degradation of tryptophan by the use of oxygen to synthesize the nicotinic acid moiety NAD+. Since the synthesis is oxygen-dependent, the yeast cells are nicotinate auxotroph in anaerobic conditions. Therefore, nicotinate must be supplied to the anaerobic growth media^28^. This behavior is captured by RPA^m^ by reporting a change associated with the upper part of kynurenine pathway till 2-amino-3-carboxymuconate semialdehyde. PADOG identified three pathways for the cut-off level of 0.01, all belonging to fatty acid degradation. Heme biosynthesis was captured at a lower significance level (0.03). Other related pathways such as glycolysis, respiration and TCA cycle were predicted to be not affected by PADOG from the oxygen availability.

### Reporter Pathways for a Disease Perturbation

Finally the two alternative approaches were applied to Alzheimer’s disease dataset^29^. The metabolite-centric reporter pathway analysis of this dataset was already reported before^30^ based on iMS570 brain metabolic model and the related manual pathway assignments, and it revealed a number of disease-related pathways which were verified from the literature data. Here, I analyze the dataset for BioCyc-based human metabolic network and with also the reaction-centric reporter pathway analysis to allow a comparison (Table 3).

Results for RPA^m^ include a number of lipid related pathways: triacylglycerol, CDB-diacylglycerol, phosphatidylcholine, 3-phosphoinositide, sphingosine and myo-inositol metabolisms. Significantly less phosphoinositides and significantly elevated myo-inositol levels were reported in Alzheimer’s disease^31^,^32^. TCA cycle is a reporter pathway based on RPA^m^, but RPA^r^ fails to capture the perturbation of this pathway in Alzheimer’s disease. It was reported that a number of mitochondrial enzymes that function in TCA cycle exhibits changes during the disease^33^,^34^,^35^. Similarly, the perturbation in melatonin degradation pathway is only successfully identified by RPA^m^. It is known that melatonin levels are reduced in Alzheimer’s disease patients, and melatonin can be used as a biomarker for the early stages of the disease^36^,^37^. Mevalonate pathway is the precursor of cholesterol biosynthesis, and cholesterol metabolism is known to be affected in Alzheimer’s disease^38^. The pathway is only identified by RPA^m^ analysis. Another pathway identified by RPA^m^, heparan sulphate biosynthesis, is in agreement with the literature since heparan sulphate proteoglycans are known to be linked to the pathogenesis of the disease^39^,^40^,^41^. Biosynthesis of retinol, a form of Vitamin A, was identified as a reporter pathway by RPA^m^, in accordance with the role of retinoids in late onset alzheimer’s disease^42^. The synthesis of N-acetylneuraminate, the most common sialic acid found in mammalian cells, is predicted to be affected during the disease (Table 3). Sialic acids are involved in the structure of glycosphingolipids, forming gangliosides. Gangliosides are known to be directly involved in Alzheimer’s disease contributing to the pathological conditions observed during the disease^43^,^44^. Sialic acids are also involved in the structure of O-glycans^45^, a related pathway of which was identified in the reporter list.

In general, RPA^r^ gives only two pathways with p-value lower than 0.01 whereas it is 20 pathways for RPA^m^. PADOG, on the other hand, did not give any significantly perturbed pathway for the selected significance cut-off of 0.01. However, it identified glutathione redox reactions and reactive oxygen species degradation in the top five significant pathways (p-values of 0.02 and 0.03 respectively). These pathways did not appear in the reporter pathway analyses. It is known that reactive oxygen species are indicative of oxidative stress in the cell, and neurodegenerative diseases are associated with oxidative stress^46^,^47^. The success of PADOG to capture these pathways, albeit at a lower significance level, could be attributed to its moderated t-statistics based significance algorithm.

### Analysis of Up/Down Pathway Regulation

In the reporter pathway analysis, the up/down regulation of the associated gene transcripts are not distinguished. I have modified RPA^m^ analysis such that significantly upregulated and downregulated pathways are also identified. Such an analysis can be combined with the identified significant pathways to reveal the capacity of RPA^m^ to predict the directionality of regulation. Due to the nature of the algorithm, in the up (/down) regulation analysis only up (/down) regulated genes linked to a metabolite are used to calculate a reporter metabolite score^48^. Since this leads to an information loss in the network connectivity, the results of such analysis must always be complemented with the results from the original analysis, as cautioned in an earlier study^48^.

RPA^m^ results for only up-regulated genes and for only down-regulated genes reveal that a high majority of the identified pathways for the genetic perturbation (Table 1) are significantly down-regulated. This is in line with the observation reported by the authors who generated the transcriptome data. They state that genes linked to NADPH-dependent reactions are commonly downregulated in the deletion strain^14^. For the environmental perturbation, the analysis identifies pathways for heme biosynthesis, glycolysis and zymosterol biosynthesis as upregulated in anaerobic conditions. High glycolytic pathway fluxes were reported in anaerobic conditions^18^. On the other hand, since zymosterol is used as a supplement in anaerobic conditions, no intracellular biosynthesis would be expected in these conditions. Such a contradiction is also available for the directionality of heme biosynthesis. The analysis identifies aerobic respiration pathways as downregulated in anaerobic condition as expected. Fatty acid oxidation pathway and the degradation of heptadecenoyl-CoA and oxtadecadienoyl-CoA are all predicted to be downregulated as reported in literature^22^. For the Alzheimer’s disease, the verification of the directionality predictions for the pathways is more challenging due to the molecular complexity of the perturbation. TCA Cycle was predicted to be down-regulated in the directionality analysis. However, some of the enzymes of the cycle were reported to be significantly increased in the disease whereas some others decreased while the rest showed no change. Melatonin degradation pathway was predicted to be up-regulated, in agreement with the reduced melatonin levels in the disease^36^. On the other hand, the calculated direction for retinol biosynthesis is upregulation whereas it is known that retinol levels diminish in Alzheimer’s disease^42^. In summary, the directionality-incorporated version of RPA^m^ can correctly predict some of the pathway regulations whereas contradictory results with the literature data were also obtained.

## Conclusions

In summary, a new metabolite-centric reporter pathway analysis was suggested as alternative to the reaction-centric approach on which a number of methods appeared recently in the literature. The power of metabolite-centric approach lies on the fact that all reactions consuming or producing the metabolites of a pathway are considered in the calculation of the pathway activity although usually such reactions are catalogued as members of other pathways, and not accounted for, in the classical approach. The direct effect of such reactions on the pathway activity is obvious although this has been mainly neglected in the pathway activity calculations. The power of metabolite-centric approach was demonstrated on three different types of perturbation; a genetic perturbation in the yeast *S. cerevisiae* due to the deletion of GDH1 gene encoding NADPH-dependent glutamate synthesis, an environmental perturbation in the yeast due to the availability of oxygen, and a disease-based perturbation due to Alzheimer’s disease. The reaction-centered analysis leads to fewer significant pathways in general compared to the metabolite-centered analysis, presenting the ability to identify the network-wide effect of perturbations of the latter. Many pathways, which were captured by RPA^m^ and known to be directly related to the phenotype of the discussed perturbations could not be identified by RPA^r^. Considering the reported power of approaches which are based on network connectivity, the success of metabolite-centric pathway identification lies on the fact that it brings the consideration of the connectivity by the reaction-centric approaches to a new level.

There are, on the other hand, a few issues to be considered for the metabolite-centric scoring approach. The tendency of the approach to report noticeably more significant pathways compared to RPA^r^ may lead to the inclusion of pathways not specific for the studied perturbation. The risk of non-specific pathways can be compensated by employing a lower cut-of for the RPA^m^ results (eg. 0.005 rather than 0.01). A more stringent cut-off of 0.005 can still capture pathways which cannot be captured by RPA^r^ for a significance level of 0.01. This includes myo-inositol, retinol and TCA cycle pathways for Alzheimer’s disease and pentose-phosphate, 2-ketoglutarate dehydrogenase complex and glycolysis pathways for the GDH1-based genetic perturbation. One should note that RPA^m^ and RPA^r^ results have different rankings for different pathways. That is, what RPA^m^ does is more than merely decreasing the cut-off. For example, mevalonate pathway, ranked as 17^th^ most significant pathway for Alzheimer’s disease based on RPA^m^, ranks as 41^th^ pathway for RPA^r^. One reason behind the change in rankings in addition to the inclusion of inter-pathway reactions is the implicit assignment of an increased weight on branch points by RPA^m^. The score of a gene is accounted multiple times in the metabolite-centered scoring especially if the metabolites of the pathway are on branch points. Although this may seem a disadvantage of the approach at first sight, the increased weight is in agreement with the fact that the branch points act as important regulatory spots in metabolism^49^,^50^, and RPA^m^ better reflects this phenomenon.

## Methods

### Metabolic Networks and Transcriptome Data

Metabolic networks for human and the yeast *Saccharomyces cerevisiae* were downloaded from BioCyc database^10^ in January 2015. Human metabolic network included 2237 metabolites and 2036 unique reactions controlled by 2521 genes while the yeast metabolic network covered 861 metabolites and 851 unique reactions controlled by 693 genes. The downloaded data also include the corresponding pathway information for each reaction. The pathways with at least three associated reactions were reported in the results. In total, there are 133 pathways for human, and 158 pathways for the yeast meeting this criteria. The networks were downloaded using SmartTables functionality of the BioCyc website, and parsed in MATLAB 2013a for further analysis.

Three different transcriptomic datasets were analyzed: the genetic perturbation in the yeast via the deletion of NADPH-dependent GDH1 gene grown in anaerobic chemostat cultures^14^, the environmental perturbation from aerobic to anaerobic condition in glucose-grown chemostat cultures of the yeast^17^, and the disease perturbation in human brain due to Alzheimer’s disease^29^. The corresponding datasets were downloaded from Gene Expression Omnibus (GSE26927, GSE4807) or obtained from the authors.

### Reporter Pathway Analysis

Reporter pathway analyses were performed using the online BIOMET Toolbox server^51^. Specifically, the *Reporter Features* tool under the online tools were used. The three input files, p-value data file, interaction network file, and network nodes-data association file, were created in MATLAB in the requested format for each specific simulation. In the metabolite-centric reporter pathway analysis (RPA^m^), a metabolite-score must be computed first. This scoring method, termed reporter metabolite analysis, was used in a number of research covering microorganisms^7^,^52^ and health problems such as liver diseases, obesity, autism^53^,^54^,^55^. Different versions of the approach also appeared^48^,^56^. For the reporter metabolite analysis, the transriptome-data based p-values were calculated by using two-sample t-test via MATLAB’s *ttest2* function. The metabolic networks downloaded from BioCyc include the information on reaction-gene association. In the analyses, the genes are linked to the corresponding controlled reactions at first as follows: when a reaction is associated with multiple genes, the one with the minimum p-value was considered^8^. Then, each metabolite in the metabolic network of interest is scored via BIOMET Toolbox server based on the neighbor genes (reactions).


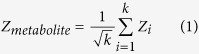


Here, Z_i_ is the Z-score of gene *i* obtained from the corresponding p-value by using the inverse normal cumulative distribution, and *k* is the number of neighbor genes to the metabolite. The averaging of the mRNA-level changes in the form of Z-scores shows the average effect of perturbation on metabolites^7^,^8^. The calculated Z-scores are corrected by the toolbox for the background distribution by using the mean (*μ*_*k*_) and standard deviation (*σ*_*k*_) of the aggregated Z scores of many sets of randomly selected k genes from the metabolic network.


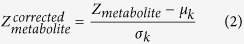


**Figure 1:**
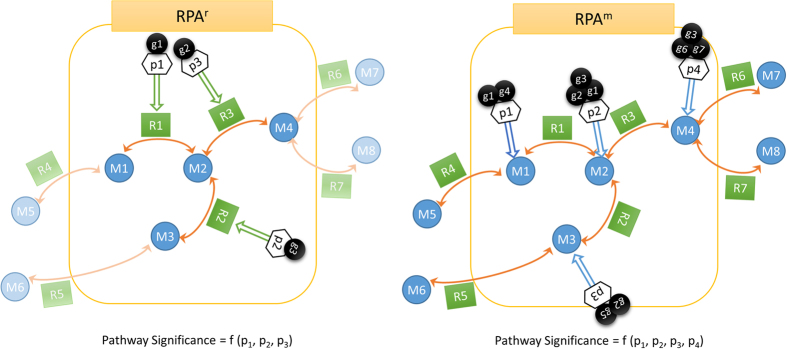
Comparison of reaction-centric and metabolite-centric reporter pathway analysis approaches. While the change in the gene activities corresponding to reactions R4, R5, R6 and R8 are not counted in RPA^r^ (reaction-centric reporter pathway analysis) since they are categorized under other pathways, they are considered in RPA^m^ (metabolite-centric reporter pathway analysis) since the metabolites of the pathway are interdependent with the rates of these reactions. (M_i_: metabolites, R_i_: reactions, g_i_: genes, p_i_: p-values).

Metabolites with significant Z-scores are called reporter metabolites, hence the analysis is termed reporter metabolite analysis^7^. Afterwards, each pathway is scored based on the involved metabolites, which were linked to the differential transcriptome data via the reporter metabolite analysis (Fig. 1). The metabolite-centric scoring of pathways (RPA^m^) follows a similar equation to eqn. (1).


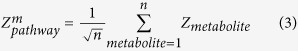


Here, *n* is the number of metabolites associated with the pathway. The output is a set of significantly affected metabolic pathways. The alternative reaction-centric reporter pathway analysis (RPA^r^) is directly based on the involved reactions in a pathway, which are linked to transcriptome data via the genes controlling the reactions. Pathways are scored via the p-values (Z-scores) of the genes associated with each involved reaction in this case, following Eqn. (4) and (5) (Fig. 1).


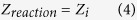



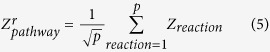


Here, Z_i_ is the score of gene *i*, and p is the number of reactions associated with the pathway. Note that both 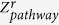 and 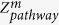 scores were corrected for the background distribution as shown in Eqn. (2). A significance level of 0.01 was used as cut-off in the analysis of the results to identify reporter pathways. Benjamini-Hochberg corrected p-values for a cut-off of 0.05 roughly corresponds to the same significant pathways for RPA^m^. The metabolite-centric approach allows the elucidation of the global effect of the perturbation on cellular parts since this scoring considers the fact that a metabolite is consumed or produced by a number of reactions which are traditionally listed under different pathways. Such an analysis bridges different cellular processes and also reflects cross-talks between pathways.

### Pathway Analysis with Down-weighting of Overlapping Genes (PADOG)

PADOG^57^ was used to compare the results of the RPA^m^ and RPA^r^ analyses. PADOG was chosen since it was shown to be one of the best performers among more than 15 alternative gene-set based algorithms^4^. The key point in PADOG is it down-weights the weight of a gene in a pathway if it is involved in multiple pathways, prioritizing the effect of pathway-specific genes in the scoring. Pathway scores are calculated by first calculating Bayesian-model based moderated t-scores for the genes^58^, and then computing the average of weighted sum of the absolute moderated t-scores of the genes in a pathway. A permutation-based test is used afterwards to assign p-values to the pathways. The related calculations were performed in the R environment, by using the PADOG package.

## Additional Information

**How to cite this article**: Çakır, T. Reporter pathway analysis from transcriptome data: Metabolite-centric versus Reaction-centric approach. *Sci. Rep.* **5**, 14563; doi: 10.1038/srep14563 (2015).

## Figures and Tables

**Table 1:** Significantly perturbed pathways for GDH1 deletion in *S. cerevisiae*.

Pathway	Number of metabolite neighbors	p-value (RPA^m^)	Number of reaction neighbors	p-value (RPA^r^)
superpathway of leucine, valine, and isoleucine biosynthesis	24	3.44E-06	6	1.75E-02
superpathway NAD/NADP - NADH/NADPH interconversion	19	8.36E-06	5	4.62E-03
leucine biosynthesis	12	9.95E-06	3	8.27E-03
pentose phosphate pathway	14	2.98E-05	6	4.36E-02
glyoxylate cycle	13	3.23E-05	5	3.67E-02
TCA cycle, aerobic respiration	23	4.70E-05	9	8.84E-03
NAD/NADP-NADH/NADPH cytosolic interconversion	15	9.41E-05	4	7.27E-03
threonine degradation	9	2.08E-04	3	2.04E-02
dolichyl-diphosphooligosaccharide biosynthesis	23	3.08E-04	11	**0.07**
gluconeogenesis	21	5.11E-04	11	**0.11**
citrulline biosynthesis	23	6.11E-04	7	**0.44**
valine degradation	10	6.20E-04	3	4.15E-02
superpathway of heme biosynthesis	18	8.31E-04	8	**0.11**
chitin biosynthesis	33	8.82E-04	14	1.91E-02
isoleucine degradation II	10	1.06E-03	3	4.15E-02
isoleucine degradation	10	1.06E-03	3	4.15E-02
pentose phosphate pathway (non-oxidative branch)	7	1.39E-03	4	**0.15**
tetrapyrrole biosynthesis II	11	1.47E-03	4	**0.24**
tetrapyrrole biosynthesis	11	1.47E-03	4	**0.24**
isoleucine biosynthesis	13	1.52E-03	4	**0.20**
glycolysis III (glucokinase)	18	1.79E-03	10	**0.10**
superpathway of serine and glycine biosynthesis I	14	2.00E-03	4	**0.33**
glycolysis	17	2.47E-03	9	**0.16**
2-ketoglutarate dehydrogenase complex	10	2.48E-03	3	**0.13**
homocysteine and cysteine interconversion	8	2.58E-03	3	**0.16**
folate transformations	20	2.65E-03	7	**0.13**
leucine degradation III	10	3.73E-03	3	**0.09**
leucine degradation	10	3.73E-03	3	**0.09**
NAD/NADP-NADH/NADPH mitochondrial interconversion	12	4.01E-03	3	1.75E-02
serine biosynthesis from 3-phosphoglycerate	11	5.15E-03	3	**0.47**
octanoyl-ACP biosynthesis (mitochondria)	20	5.31E-03	6	**0.39**
superpathway of allantoin degradation in yeast	13	5.36E-03	4	**0.10**
valine biosynthesis	12	5.53E-03	4	**0.20**
lysine biosynthesis	22	7.47E-03	6	**0.34**
folate interconversions	22	7.58E-03	8	**0.23**

Pathways with RMA^m^ p-value lower than 0.01 are listed. RPA^r^ p-values greater than 0.05 are given in bold for a better demonstration of pathways not captured by RPA^r^.

**Table 2:** Significantly perturbed pathways for aerobic-anaerobic change in *S. cerevisiae*.

Pathway	Number of metabolite neighbors	p-value (RPAm)	Number of reaction neighbors	p-value (RPAr)
10-trans-heptadecenoyl-CoA degradation (reductase-dependent)	19	6.02E-08	5	1.06E-03
10-cis-heptadecenoyl-CoA degradation	19	9.89E-08	5	1.06E-03
9-cis, 11-trans-octadecadienoyl-CoA degradation (isomerase-dep.)	18	2.97E-07	4	2.09E-03
aerobic respiration (cytochrome c)	13	1.69E-05	4	8.71E-03
aerobic respiration (linear view)	13	1.69E-05	4	8.71E-03
fatty acid oxidation (non-cyclic)	18	5.23E-05	5	6.59E-04
fatty acid beta-oxidation (peroxisome)	16	1.31E-04	4	5.03E-03
tryptophan degradation VIII (to tryptophol)	10	1.96E-04	3	2.56E-03
gluconeogenesis	21	2.38E-04	11	2.71E-03
glycolysis III (glucokinase)	18	3.47E-04	10	3.83E-03
glycolysis	17	4.39E-04	9	8.73E-03
glyoxylate cycle	13	6.54E-04	5	7.01E-03
sphingolipid biosynthesis	24	8.04E-04	8	2.60E-02
ethylene biosynthesis	12	1.10E-03	3	4.79E-02
oleate beta-oxidation (reductase-dependent)	7	1.16E-03	3	1.69E-02
superpathway phosphatidate biosynthesis (yeast)	12	2.04E-03	4	**0.19**
superpathway of leucine, valine, and isoleucine biosynthesis	24	2.40E-03	6	**0.12**
superpathway NAD/NADP - NADH/NADPH interconversion	19	3.17E-03	6	**0.08**
zymosterol biosynthesis	22	3.89E-03	5	**0.07**
phenylalanine degradation	12	4.00E-03	4	3.52E-03
heme biosynthesis	11	4.13E-03	4	1.59E-03
pentose phosphate pathway	14	5.25E-03	6	**0.38**
TCA cycle, aerobic respiration	23	5.38E-03	9	**0.07**
chitin degradation to ethanol	20	6.78E-03	6	8.63E-03
NAD/NADP-NADH/NADPH cytosolic interconversion	15	7.98E-03	4	**0.23**
tryptophan degradation to 2-amino-3-carboxymuconate semialdehyde	13	8.19E-03	5	1.25E-02

Pathways with RMA^m^ p-value lower than 0.01 are listed. RPA^r^ p-values greater than 0.05 are given in bold for a better demonstration of pathways not captured by RPA^r^.

**Table 3:** Significantly perturbed pathways for Alzheimer’s disease.

Pathway	Number of metabolite neighbors	p-value (RPAm)	Number of reaction neighbors	p-value (RPAr)
triacylglycerol biosynthesis	10	1.58E-06	4	5.10E-03
terminal O-glycans residues modification	19	1.97E-05	7	2.38E-02
pyrimidine deoxyribonucleotides *de novo* biosynthesis	19	7.20E-05	5	**0.06**
CDP-diacylglycerol biosynthesis	12	8.87E-05	4	**0.09**
phosphatidylcholine biosynthesis	11	1.59E-04	3	**0.19**
D-myo-inositol (1,4,5)-trisphosphate biosynthesis	12	1.79E-04	4	4.38E-02
pyrimidine deoxyribonucleotides biosynthesis from CTP	17	2.51E-04	5	**0.06**
chondroitin sulfate biosynthesis (late stages)	12	4.66E-04	4	6.82E-03
retinol biosynthesis	15	2.00E-03	6	**0.08**
3-phosphoinositide biosynthesis	15	2.15E-03	7	2.74E-02
phospholipases	11	2.17E-03	4	**0.15**
gluconeogenesis	29	2.78E-03	12	**0.17**
TCA cycle	31	3.53E-03	8	**0.13**
CMP-N-acetylneuraminate biosynthesis I (eukaryotes)	16	5.16E-03	4	**0.17**
melatonin degradation I	15	6.81E-03	3	**0.23**
sphingosine and sphingosine-1-phosphate metabolism	9	6.99E-03	3	3.74E-02
mevalonate pathway	17	7.20E-03	7	**0.26**
4-hydroxyproline degradation	14	9.04E-03	3	**0.05**
D-myo-inositol-5-phosphate metabolism	8	9.35E-03	3	**0.36**
heparan sulfate biosynthesis (late stages)	22	9.54E-03	9	**0.10**

Pathways with RMA^m^ p-value lower than 0.01 are listed. RPA^r^ p-values greater than 0.05 are given in bold for a better demonstration of pathways not captured by RPA^r^.
